# The G protein-coupled estrogen receptor 1 (GPER/GPR30) does not predict survival in patients with ovarian cancer

**DOI:** 10.1186/1757-2215-5-9

**Published:** 2012-03-18

**Authors:** Zuzana Kolkova, Vera Casslén, Emir Henic, Sara Ahmadi, Anna Ehinger, Karin Jirström, Bertil Casslén

**Affiliations:** 1 Department of Gynecology & Obstetrics, Department of Clinical Sciences, Lund University, Skåne University Hospital Lund, SE-221 85 Lund, Sweden; 2 Division of Pathology, Department of Clinical Sciences, Lund University, Skåne University Hospital Lund, SE-221 85 Lund, Sweden

**Keywords:** ERα, ERβ, borderline tumors, TMA, immunohistochemistry, ovarian cancer cell lines

## Abstract

**Background:**

Even though ovarian tumors are not generally considered estrogen-sensitive, estrogens may still have an impact on ovarian tumor progression. The recently identified trans-membrane estrogen receptor GPER is involved in rapid estrogen signaling. Furthermore, it binds selective estrogen receptor modulators with agonistic effect, which could explain tamoxifen controversies.

**Methods:**

GPER mRNA was assayed with quantitative real-time PCR (qPCR) in 42 primary ovarian tumors and 7 ovarian cancer cell lines. ERα and ERβ mRNA were analyzed for comparison. GPER protein was semi-quantified with densitometric scanning of Western blots and its tissue distribution analyzed with immunohistochemistry (IHC) in 40 ovarian tumors. In addition, IHC was evaluated in a tissue microarray (TMA) of 150 primary malignant ovarian tumors.

**Results:**

All tumor samples contained GPER mRNA. The content of mRNA was not different between benign and malignant tumors, but one third of malignant samples over-expressed GPER mRNA. The content of ERα mRNA was higher in malignant than in benign tumors, whereas ERβ mRNA was higher in benign than in malignant tumors. GPER mRNA was detected in all seven ovarian cancer cell lines with highest levels in TOV21G and TOV112D cells. Similar expression pattern was seen for ERβ mRNA. Western blot demonstrated GPER protein in all tumor samples. Semi-quantification showed no difference between benign and malignant tumors, but about one third of malignant samples over-expressed GPER protein. GPER staining was localized mainly in epithelial cells. In the TMA study we found no correlation between GPER staining and clinical stage, histological grade or patient survival.

**Conclusions:**

GPER mRNA as well as GPER protein is present in both benign and malignant ovarian tumor tissue. About one third of malignant tumors over-expressed both GPER mRNA and protein. This, however, correlated neither with histological or clinical parameters nor with patient survival.

## Background

Epidemiologic data suggest on the one hand that estradiol taken as oral contraceptive in the premenopause decreases the risk of ovarian cancer, while taken as hormone therapy in the postmenopause increases that risk. From a clinical perspective, development and progression of ovarian tumors are not generally considered estrogen sensitive, as is the case for breast and endometrial cancer. However, some experimental data challenge this perception. Patients with ovarian tumors have elevated blood levels of estradiol, and estrogenic steroids stimulate proliferation in several ovarian cancer cell lines [1]. Also, apoptosis was reduced by estradiol in immortalized ovarian surface epithelial cells via Akt mediated up-regulation of bcl-2, an anti-apoptotic gene [2]. These effects of estradiol involved the nuclear estrogen receptor α (ERα), and distribution of ERα in malignant ovarian tumor tissue overlapped with a marker for cell proliferation as well as with lower apoptotic activity [3]. In contrast, the other nuclear estrogen receptor ERβ inhibited cell growth and induced apoptosis [4].

The two nuclear estrogen receptors ERα and ERβ are present in normal ovarian surface epithelial cells as well as in ovarian tumors and cancer cell lines. A general feature, which has been documented in numerous tumor types, including ovarian, breast, prostatic, lung and colorectal cancer is an increase of ERα and a decrease of ERβ in malignant as compared to corresponding benign tumors [5].

The transmembrane G protein-coupled receptor 30 (GPR30) was recently reported to bind estradiol with high affinity, K_D _3-6 nmol/L, i.e. 10 times higher than that of ERα [6], and was re-named G protein-coupled estrogen receptor 1 (GPER). It is localized in the cell membrane as well as in intracellular membranes [7,8]. GPER is widely expressed in the human body, both in normal and pathological tissues. In addition to estradiol, this receptor binds selective estrogen receptor modulators (SERM), e.g. tamoxifen, as well as antagonists, e.g. ICI 182780, creating an agonistic response [6]. Downstream signaling involves second messengers like MAP and PI3 kinases, as well as trans-activation of the epidermal growth factor receptor [9].

This study of GPER in primary ovarian tumors and ovarian cancer cell lines was partly initiated because SERM molecules, like tamoxifen, have come in use as adjuvant therapy in patients with ovarian cancer, and these compounds are GPER agonists. A minority of these patients, about 10-15%, responds clinically to treatment with tamoxifen, but whether this effect involves ERα, ERβ, GPER, or all of them, remains still an open question. In fact, both GPER and ERα along with an intact EGFR signaling were required for estrogen-stimulated proliferation of ovarian cancer cells [10]. In contrast, a recent paper by Gao et al. suggests that activation of epithelial GPER inhibits uterine growth by paracrine inhibition of stromal ERα signaling [11]. These observations, together with complex and tissue-specific responses to SERMs, suggest intricate interactions between the nuclear and membrane estrogen receptors.

We assayed GPER mRNA and GPER protein in benign and malignant tumors, analyzed GPER protein distribution in the tumor tissue, and also explored the possibility that GPER expression correlates with tumor histology or survival in patients with ovarian cancer.

## Methods

### Tumor tissue samples for real-time PCR, western blot and immunohistochemistry

Ovarian tumor tissue samples (n = 42) were obtained at operation, Department of Obstetrics and Gynecology, Lund University Hospital, during 2001-2007. The oncologic surgeon selected the precise area for tumor biopsies. The samples were cut in 5 × 5 × 5 mm cubes, quick frozen on dry ice, and stored at -80°C. Tumors were sent for routine histopathological examination. Histological parameters were subsequently re-evaluated by one of us (A.E.), and classification of the material is shown in Table 1. Archival paraffin embedded section were retrieved for immune histochemistry. The study was approved by the Ethical Review Board at Lund University Hospital.

**Table 1:** Histopathology of primary ovarian tumors used in qPCR and Western blot

	Serous	Mucinous	Endometrioid	Total
BENIGN	4	5		9

BORDERLINE	6	5		11

GRADE 1	6	2		8

GRADE 2		1	3	4

GRADE 3	5		5	10

Total	21	13	8	42

### Tumor tissue samples for tissue micro-array (TMA) construction

Formalin-fixed and paraffin embedded archival tissues from primary malignant epithelial ovarian tumors (n = 154) were used. All cases were histopathologically re-evaluated and tumor content verified in hematoxylin-eosin stained slides. This material and TMA construction has previously been detailed in several publications [12-16].

### Ovarian cancer cell lines

Seven human cell lines all derived from epithelial ovarian adenocarcinomas were cultured under specified conditions on uncoated plastic. TOV21G, TOV112D, SKOV-3, OVCAR-3, ES-2 were grown according to ATCC recommendations http://www.lgcstandards-atcc.org/. SKOV-3ip (a gift from Tumor Immunology, Lund University, Sweden), and HEY-TG (a gift from M.D. Anderson Cancer Institute, Houston, TX, USA) were cultured in M199 with 10% FBS as we previously described [17]. Culture media and supplements were obtained from Invitrogen, Gibco (Carlsbad, CA, USA).

### Gene expression

Total RNA was extracted from about 125 mg frozen ovarian tumor tissue. The tissue was homogenized in Trizol 50 mg/mL (Invitrogen, Carlsbad, CA) using rotating-knives (Polytron). Total RNA from harvested cells was extracted using EZNA Total RNA Kit™ (OMEGA Bio-Tec, Doraville, GA, USA). All RNA samples were evaluated for concentration and purity by NanoDrop Spectrophotometer ND-1000 (Saveen Werner, Limhamn, Sweden) as well as quality by 2% agarose gel electrophoresis. For reverse transcription to cDNA we used TaqMan Reverse Transcription Reagents (Applied Biosystems, Foster City, CA, USA). The final concentration of cDNA was 10 ng/μL (+/- 7%).

Real-time PCR was performed using ABI PRISM 7000 (Applied Biosystems) with following pre-manufactured assays (Applied Biosystems): Hs00173506_m1 (GPER), Hs00174860_m1 (ERα), Hs00230957_m1 (ERβ), Hs99999903_m1 β-actin(ACTB) and Hs99999142_m1 cyclin-dependent kinase inhibitor 1A (CDKN1A). Several reference genes were evaluated both in tumor tissue samples and in the cell lines for minimal variation between groups and cell lines. CDKN1A was chosen for the tumor samples and ACTB for the cell lines. Quantification employed a calibration curve obtained by serial dilutions of the template DNA (80 - 0.08 ng). Results are expressed as relative values.

### Western blot and semi-quantification of GPER protein

Ovarian tumor tissue (65-75 mg), HEK-293 cells (negative control), SKBr-3 and MCF-7 cells (positive controls) were disintegrated in QIAGEN TissueLyser (Retsch Technology GmbH, Haan, Germany) and membrane proteins prepared as described [18]. Each membrane fraction (20 μg total protein) was analyzed with Western blot [18]. We used the anti-human goat GPER antibody (AF5534, R&D systems Inc., Minneapolis, MN, USA). The immunogen was a 62 amino acid E. coli derived peptide, which constitutes the extra-cellular N-terminal of human GPER. The specific band was scanned (Syngene, Cambridge, UK) and the density (Gene Tools, Philomath, Oregon, USA) was taken as a semi-quantitative measure of GPER protein.

### Immunohistochemistry of GPER

Sections were de-parafinized and re-hydrated, antigen retrieved in Buffer Dako S 1699 under pressure at 121°C, and finally endogenous peroxidase blocked by Dako S 2023 solution. IHC staining was performed in Autostainer Plus (Dako A/S, Glostrup, Denmark) using as primary antibody either goat anti-GPER (AF5534) or mouse monoclonal anti-ERα antibody (M 7047, Dako) both diluted 1:50. Biotinylized anti-goat and anti-mouse antibodies (Dako) respectively, were used as secondary antibodies. A streptavidin-peroxidase complex was used for detection, and peroxidase activity visualized by Dako Real™ Detection System (K5001, Dako). Non-immune goat or mouse IgG (Dako) replaced the primary antibody as negative control.

### Semi-quantification of immuno staining in TMA slides

For assessment of GPER expression staining intensity as well as the fraction of positive cells we used a modification of a previously described semi-quantitative scoring system [19]. Staining intensity (I) was categorized as 0 (negative), 1 (weak), 2 (moderate) or 3 (strong). The fraction of positive cells (F), which took into account both membrane and cytoplasmic staining in the tumor cells, was classified as 0 (0-1%), 1 (2-10%), 2 (11-50%) and 3 (> 50%). A staining score was then created by I × F, which ranged from 0 to 9. For statistical purposes the staining score was further categorized as negative (0-1), weak (2-3), and strong (4-9). The immuno staining was assessed by two independent observers (Z.K., A.E.). The observers had minor disagreement in about 10% cases. These samples were re-evaluated by both observers in order to reach a consensus.

### Statistical methods

Data are presented as scatter box plots with median and percentiles. The Mann-Whitney and Kruskal-Wallis test were used to evaluate the significance of differences between groups. Spearman's test was used to assess the rank correlation between different mRNA assays and staining. Jonckheere's test was performed evaluate the significance of trends between groups. Fisher's exact test was used to compare number of samples with over-expression between groups. Kaplan-Meier analysis and log rank test were used to analyze differences in overall survival in patients with ovarian cancer stratified according to GPER expression. All tests were two-sided and 5% level of significance was used.

## Results

### GPER mRNA in ovarian tumors

**Figure 1:**
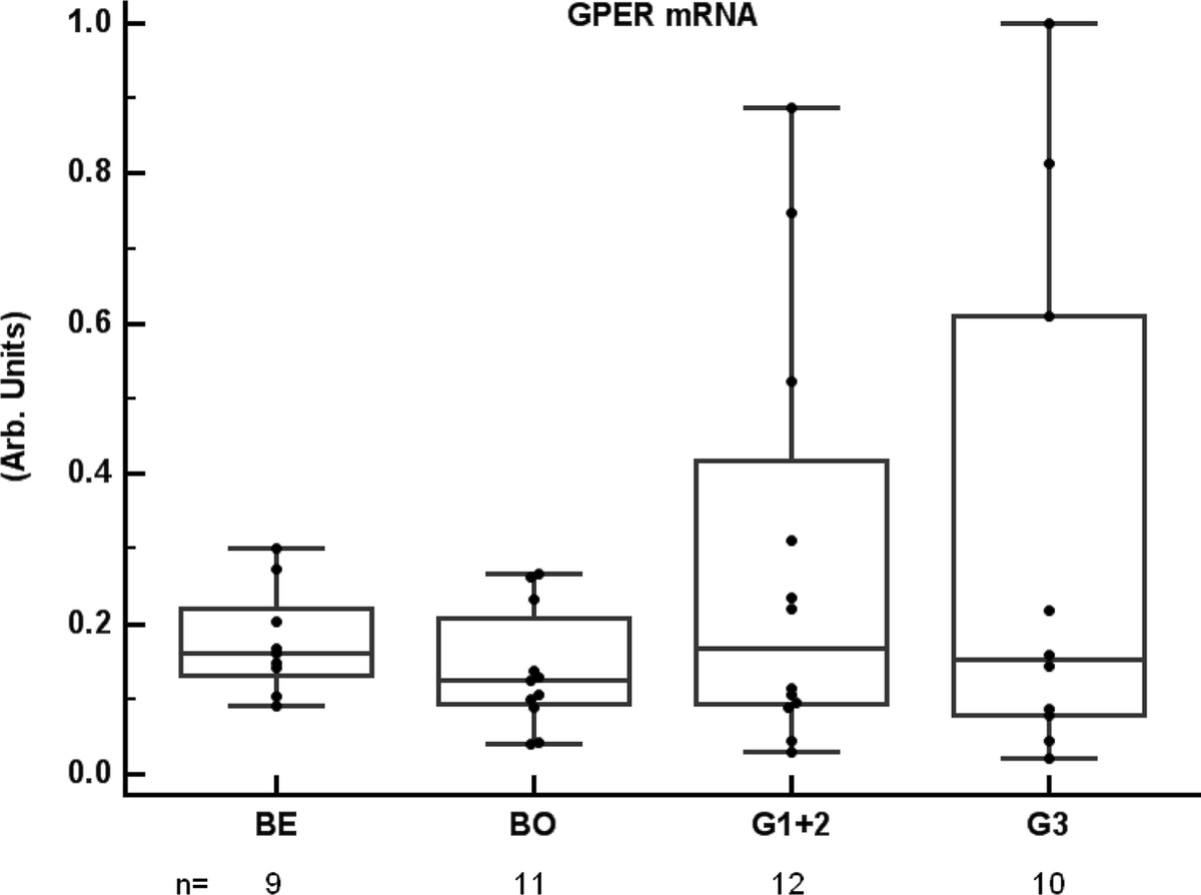
**GPER gene expression in ovarian tumors GPER mRNA, normalized to CDKN1A mRNA, in ovarian tumor tissue samples (n = 42)**. Histology was classified as benign (BE), borderline (BO), and malignant. The latter group was further split according to differentiation as grade 1 (G1), grade 2 (G2), and grade 3 (G3). G1 and G2 are presented together due to low number of samples in G2. The number of samples (n) in each group is indicated in the figure. GPER mRNA levels were not different between the groups, but the number of samples with high GPER mRNA levels (> 0.5 arbitrary cut-off) was higher in malignant (6/22) than in benign/borderline samples (0/20), (p = 0.02; Fisher's exact test).

All primary ovarian tumor samples i.e. benign, borderline and malignant, expressed GPER mRNA (Figure 1). Serous, mucinous, and endometrioid tumors are presented together, since no consistent difference in expression was found between these histological types. The level of GPER mRNA was not different between the benign/borderline and malignant groups. Furthermore, there was no relation to loss of differentiation within the malignant group. However, the number of samples expressing GPER mRNA above the arbitrary cut-off 0.5 was significantly higher in the malignant group 6/22 (27%) as compared to the benign/borderline groups 0/20 (0%).

### ERα mRNA and ERβ mRNA in ovarian tumors

**Figure 2:**
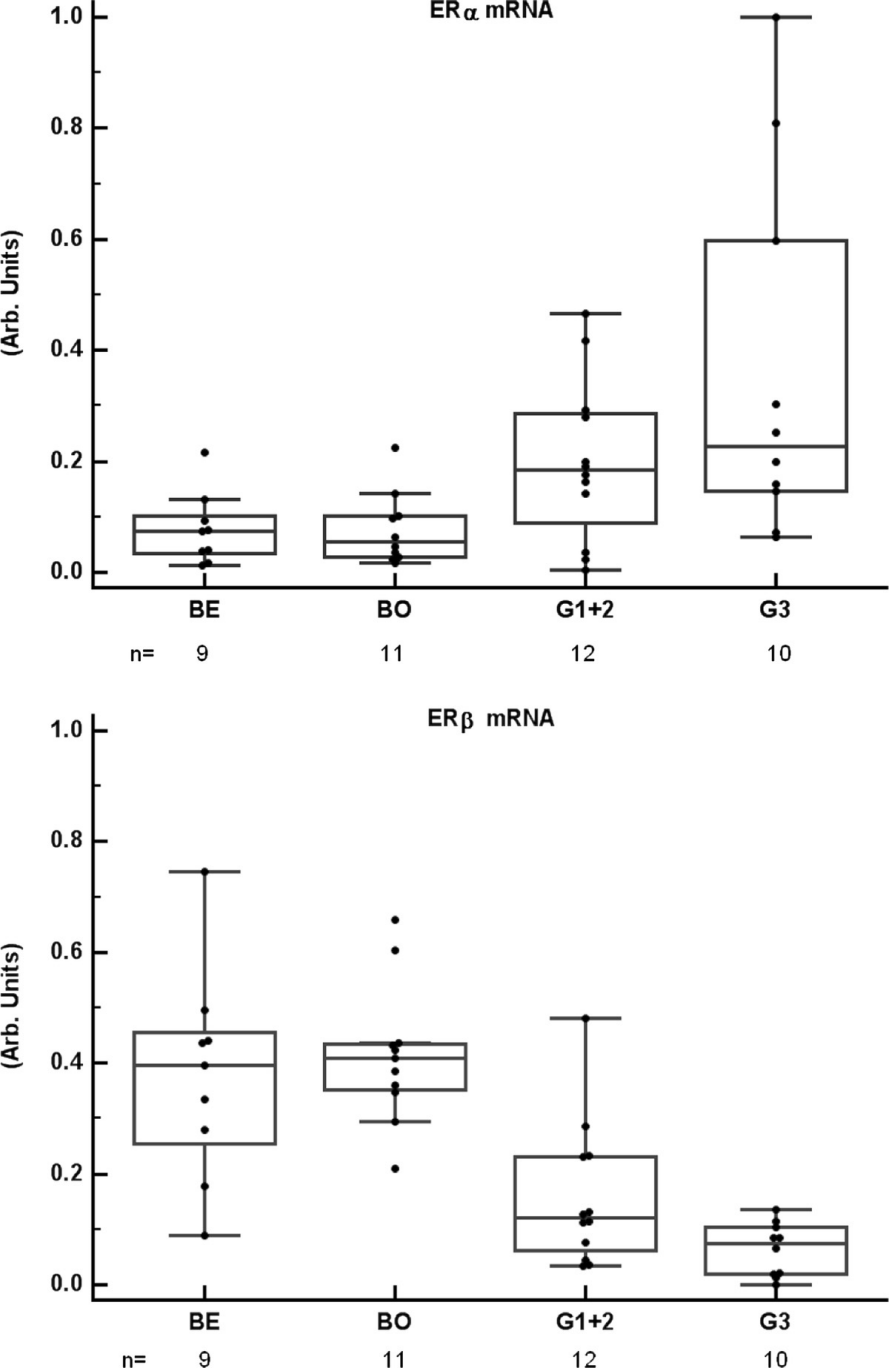
**ERα and ERβ gene expressions in ovarian tumors ERα mRNA and ERβ mRNA, normalized to CDKN1A mRNA, in the same set of tumor samples as in Figure 1**. Abbreviations are as in Figure 1. ERα mRNA was higher in malignant than in benign/borderline samples (p = 0.01), whereas ERβ mRNA was lower in malignant than in benign/borderline samples (p < 0.0001).

For comparison, the same set of samples was analyzed for ERα and ERβ mRNA (Figure 2). The content of ERα mRNA was higher in truly malignant tumors than in benign/borderline tumors, whereas the content of ERβ mRNA was lower in malignant than in benign/borderline tumors. These divergent patterns of the nuclear ERs were unlike that of GPER mRNA. However, 5 out of 6 GPER high-expressers had also high levels of ERα mRNA. GPER mRNA and ERα mRNA showed a weak correlation (rho = 0.5; p = 0.001).

### GPER, ERα and ERβ mRNA in ovarian cancer cell lines

**Figure 3:**
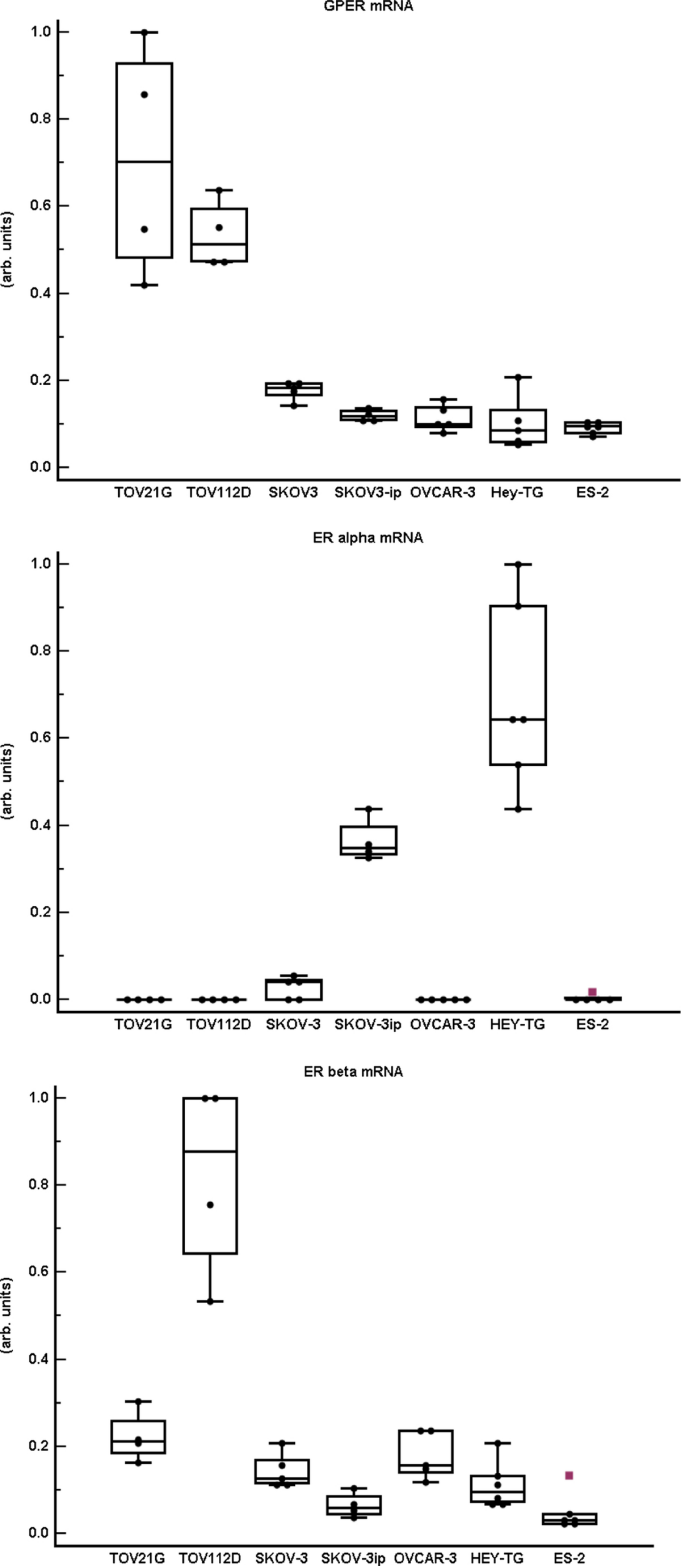
**GPER, ERα and ERβ gene expression in ovarian cancer cell lines GPER, ERα, and ERβ mRNAs were normalized to corresponding β-actin mRNA in seven ovarian cancer cell lines (analyzed in 4-6 wells)**. Cell lines are arranged in order of declining GPER mRNA level. All cell lines expressed GPER mRNA and ERβ mRNA with highest levels in TOV21G and TOV112D. ERα mRNA was not detected in TOV21G, TOV112D, OVCAR3, ES2, and in insignificant amounts in SKOV-3.

All seven ovarian cancer cell lines expressed GPER mRNA. Highest expression was seen in TOV-112D and TOV-21 G, whereas the other five cell lines expressed at a lower level (Figure 3). ERα mRNA and ERβ mRNA were analyzed for comparison. All seven cell lines expressed ERβ mRNA, and the pattern had similarities to that of GPER mRNA with high expression mainly in TOV-112D but also in TOV-21 G (Figure 3). In contrast, ERα mRNA was detected at a significant level in only two of the cell lines, SKOV-3ip and HEY-TG (Figure 3).

### GPER protein in ovarian tumors

**Figure 4:**
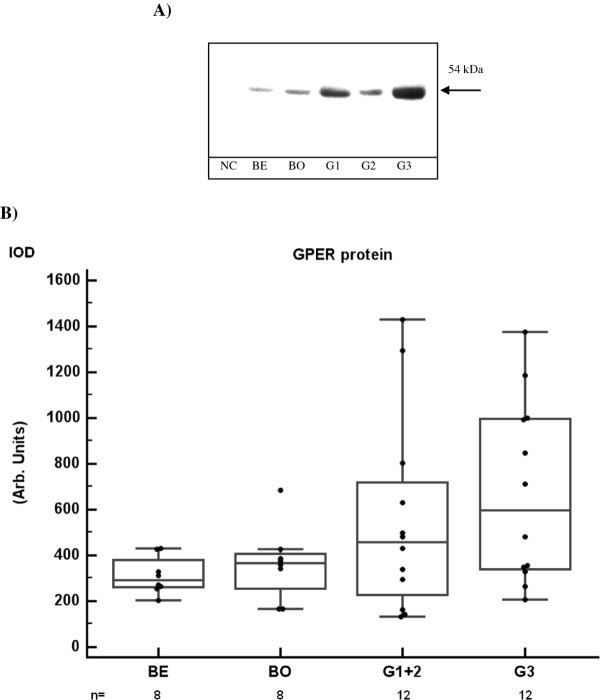
**Western blot of GPER in ovarian tumors with semi-quantification A) GPER protein was detected as a single band at 54 kDa in Western blots of primary ovarian tumor extracts**. Abbreviations are as in Figure 1. NC = negative control (extract from HEK-293 cells). **B) **The tissue content of GPER protein was semi-quantified by densitometric scanning of the bands, and presented as integrated optical density (IOD). The number of samples (n) in each group is given in the graph. GPER protein levels were not different between benign/borderline tumors and malignant tumors, although G3 tumors had higher level than benign tumors (p = 0.03). However, the number of samples with elevated GPER protein (> 500 arbitrary cut-off) was higher in malignant (10/24) than in benign/borderline tumors (1/16), (p = 0.01, Fisher's exact test).

Western blot detected GPER protein as a single band of varying intensity at 54 kDa in all primary ovarian tumors (Figure 4A). In addition to the samples used for qPCR, another two grade 3 tumors could be included in this analysis. GPER protein was semi-quantified by densitometric scanning of the band for each sample, and results are presented according to histological differentiation (Figure 4B). We found no significant difference between the malignant group and the benign/borderline group (Mann-Whitney test, Kruskal-Wallis).

However, the malignant tumors showed greater variation in the tissue content of GPER protein than did the benign/borderline tumors. In fact, the number of samples with GPER above the arbitrary cut-off level 500 was significantly higher in the malignant group 10/24 (42%) than in the benign/borderline group 1/16 (6%). High expressing samples appeared in all three malignant grades groups, suggesting no relation to loss of histological differentiation.

### Tissue distribution of GPER protein in ovarian tumors

**Figure 5:**
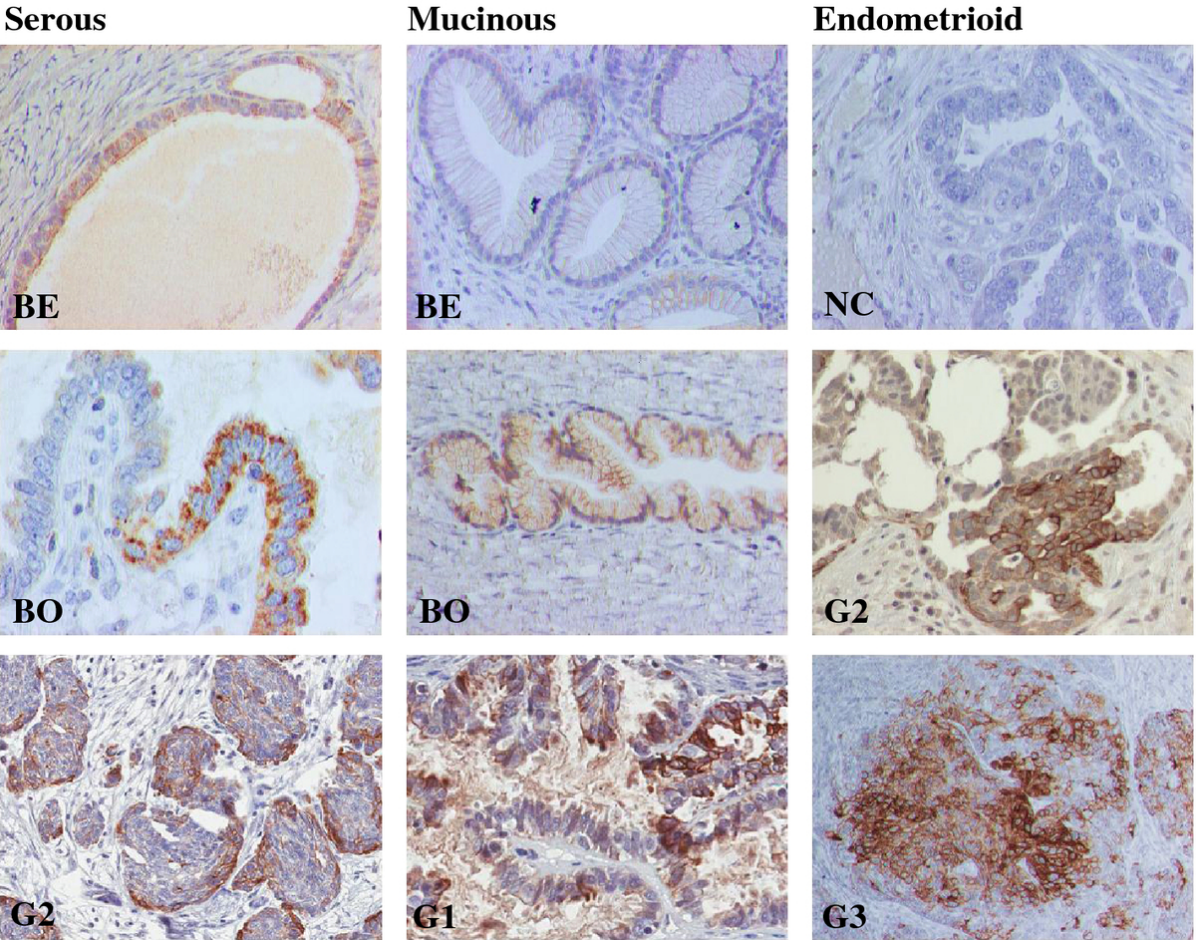
**Distribution of GPER immunohistochemical staining in serous, mucinous and endometrioid ovarian tumors Altogether 37 samples were evaluated**. Abbreviations are as in Figure 1. Non-immune IgG replaced the GPER antibody as a negative control (NC). Staining was mainly localized in tumor cells, but also in single stromal cells. It tended to be strongest in tumor cells close to the stroma. Staining could be identified both in cell membranes and in the cytoplasm.

IHC analysis revealed that GPER was localized mainly to epithelial tumor cells (Figure 5). Staining was focal and often strongest in tumor cells close to the stroma. Some malignant samples had stronger staining intensity than benign and borderline samples. Both membrane and cytoplasmic staining was observed in the tumor cells. Staining was also noted in single, possibly migratory, cells in the stroma.

### Semi-quantification of GPER in TMA and relation to clinical outcome

**Figure 6:**
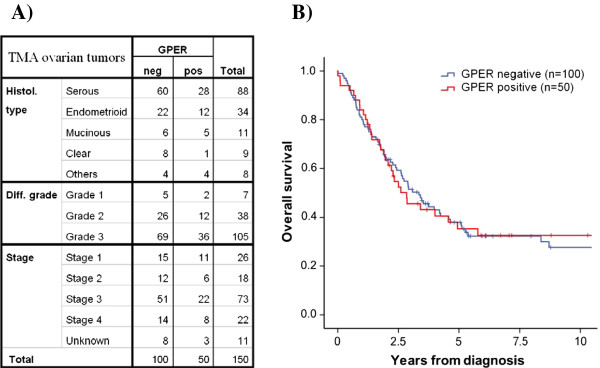
**Tumor tissue content of GPER related to histological and clinical parameters TMA of 150 malignant primary ovarian tumors were immunostained for GPER**. **A) **Each histological type and grade as well as clinical stage was stratified as GPER positive samples (score 2-9) vs. GPER negative samples (score 0-1). The distribution of GPER positive samples was not different between histological types, differentiation grades, or clinical stages. **B) **Kaplan-Meier curves of overall survival for patients showed no difference between GPER positive and GPER negative tumors.

GPER expression could be evaluated in 150/154 (97%) primary tumors. Evaluation included only staining in tumor cells. According to our staining score 100 samples (67%) were negative (score 0-1), 27 samples (18%) showed weak positivity, and 23 (15%) strong positivity (Figure 6A). Positive GPER staining correlated neither with histological grade or type nor with clinical stage. Furthermore, positive staining was not predictive for overall survival of the patients (Figure 6B). Also, staining intensity and positive cell fraction, when evaluated separately, lacked correlation with the histological and clinical parameters. In order to verify sensitivity and specificity of the staining process, a TMA slide with 50 malignant breast tumors (kindly provided by Dept. of Oncology, Lund, Sweden) was stained for GPER. Applying the same evaluation criteria, 28 samples (56%) were positive. In addition, we found that 25/50 (50%) GPER positive tumors were also positive for ERα, but the correlation coefficient was not significant.

## Discussion

This is the first study that quantifies GPER expression in benign, borderline, and malignant ovarian tumors using qPCR and Western blot. One study, which previously described GPER expression in ovarian tumors [20], used IHC as the only method. GPER expression has also been reported in cell lines derived from various reproductive organ tumors, like ovarian cancer [10].

Since, apparently, about one third of malignant samples showed high GPER expression in qPCR and Western blot, we wished to explore the possibility that this third might have a different clinical outcome than the low-expressing two thirds. In our prospective population-based TMA study of 150 malignant primary tumors, one third of the tumors had significant staining. However, this third was not distinguished from the negative samples by any of the clinical parameters i.e. stage of the disease, histological grade, and over-all survival. This finding disagrees with the results of Smith et al., who reported that high GPER expression was associated with high histological grade and clinical stage as well as poor survival in an IHC study of 89 malignant and 45 borderline ovarian tumors [20]. Furthermore, another two IHC studies observed correlation between GPER and unfavorable clinicopathological features. One study with a large number of malignant breast tumors showed that over-expression of GPER protein was associated with poor prognostic parameters like large tumor size, distant metastases, and over-expression of HER2 [21]. The other study on primary endometrial cancer reported that over-expression of GPER protein in the tumor tissue correlated with poor differentiation, aggressive subtype, and advanced clinical stage [22]. However, in this context it should also be noted that another study of GPER mRNA in breast cancer tissue failed to identify any correlation between the level of expression and clinical parameters [23].

Generally, discrepancies between immunohistochemical studies may relate to different populations studied, tissue handling and processing, specificity and sensitivity of the primary antibody, characteristics of the detection system, criteria used in the evaluation process, etc. We employed the GPER antibody we considered most reliable among those commercially available and used it in two methods. In Western blot, this antibody gave one band in all tumor samples. Densitometric scanning found the band to be strong in about one third of the samples. In IHC, our evaluation of TMA found roughly one third of samples positive. Thus, our Western Blot and IHC results revealed similar fraction of high-expressing/positive samples. In addition, these data derive support from our qPCR results. Comparison with other methods was not done in previously mentioned IHC studies.

To check sensitivity of the antibody, we included a TMA slide with 50 breast tumors in a control experiment. After an identical staining process we found that about 60% of the samples were positive, which is comparable to that reported in previous study of breast cancer [21]. Hence the fraction of GPER positive samples was smaller in ovarian tumors than in breast tumors. In fact, GPER seems to be involved in both proliferative and anti-proliferative effects, which are tissue specific [24-26].

We found that GPER immune staining was mainly localized in the malignant epithelial cells, although focal weak staining as well as single cell staining was also present in the stroma. This distribution matches above-mentioned IHC studies of reproductive malignancies, and it is also similar to the GPER distribution we and others previously reported in normal human endometrial tissue [11,18]. Even though epithelial distribution of GPER is a common feature in female reproductive organs and their tumors, GPER is also expressed in other cell types in these organs. In the malignant cells, we found GPER staining both in the plasma membrane and in the cytoplasm. This is in agreement with a previous study, which identified intracellular GPER trafficking between the plasma membrane and cytokeratin intermediate filaments [8]. Interestingly, Smith et al. did not report membrane staining, but found nuclear staining together with cytoplasmic staining [20].

Expression of the two nuclear estrogen receptors had opposite patterns in ovarian tumors, i.e. ERα mRNA was higher whereas ERβ mRNA was lower in malignant tumors, while the reverse relation was seen in benign tumors. In fact, our results are supported by similar findings in a previous mRNA study of ERα and ERβ in ovarian tumors [27], and this seems to adhere to the general principle that ERα increases and ERβ decreases with loss of histological differentiation. The expression of GPER mRNA had a different pattern than that of ERα mRNA and ERβ mRNA since it showed no significant difference between the benign, borderline and malignant tumors. However, there was a weak correlation between GPER and ERα mRNA. Such similarity between these mRNA expressions, which we previously observed in normal human endometrium [18], has also been reported in breast cancer [21,23,28]. Interestingly, GPER and ERβ had very similar pattern of mRNA expression in the seven ovarian cancer cell lines, whereas ERα mRNA was expressed in only three out of seven ovarian cancer cell lines, and only at a low level.

Proliferation in ovarian cancer cells is influenced by estrogen. BG-1 ovarian cancer cells, which express both GPER and ERα, respond to both estradiol and to a selective GPER agonist G-1 with induced expression of c-fos and cyklins D1, E, and A [10]. Noticeably, both GPER and ERα were needed for the response, also when cells were stimulated with G1. Furthermore, inhibition of the EGFR transduction pathway inhibited c-fos stimulation and ERK activation by both ligands, supporting previous reports that GPER activation and signaling involves trans-activation of the EGFR [9]. GPER may thus play a role in cancer cell proliferation possibly through trans-activation of EGFR, or as an ERα collaborator. However, GPER is also known to mediate ERα antagonizing effects depending on the cell/tissue type [29].

GPER is further involved in ovarian cancer cell migration. Using a trans-membrane migration assay, we found that EGF stimulated migration in seven ovarian cancer cell lines (same as used in this study), and that this resulted from increased cell surface expression of ligated uPAR. In contrast to Park et al. [30], we did not find a direct effect of estradiol on migration in any of the seven ovarian cancer cell lines [31]. However, estradiol attenuated the stimulatory effect of EGF on migration in all seven cell lines [31] through inhibition of EGF-induced accumulation of detergent extractable uPAR. Furthermore, since Tamoxifen and ICI 182780, antagonists to nuclear ERs, and G-1, a specific GPER agonist, mimicked this effect of estradiol, we concluded that estrogen modulation of EGF induced migration in ovarian cancer cells was mediated by GPER, not by ERα.

Since expression of the EGF system in ovarian tumors relates to poor prognosis as well as to poor response to chemotherapy [32,33], and estradiol mediated activation of GPER attenuates the invasive properties resulting from EGF stimulation [31], a GPER agonist could have therapeutic implications in patients with ovarian cancer. On the other hand, our present study shows that GPER is neither a suitable diagnostic marker, since only one third of malignant ovarian tumors have increased expression, nor a prognostic marker, as it does not correlate with patient outcome. Finally, GPER most likely contributes to complexity of the clinical response to SERMs, like tamoxifen, and also function as a co-player to the nuclear ERs.

## Conclusions

GPER expression, both at the mRNA and protein level, was detectable in all tissue samples of benign, borderline and malignant ovarian tumors. We also report that the tumor tissue content of neither GPER mRNA nor the protein was different between benign and malignant tumors, although both the mRNA and the protein were over-expressed in about one third of the malignant tumors. However, this GPER positive third of malignant ovarian tumors had no relation to clinical parameters.

## Abbreviations

GPER: GPR30, G protein-coupled estrogen receptor, G protein-coupled receptor 30; ERα: Estrogen receptor α; ERβ: estrogen receptor β; qPCR: quantitative real-time PCR; TMA: Tissue Microarray; IHC: immunohistochemistry; OS: overall survival.

## Competing interests

The authors declare that they have no competing interests.

## Authors' contributions

ZK carried out the Western Blot analysis, part of the gene expression experiments, evaluation of the immunohistochemical staining, statistical analysis, and drafted the manuscript. VC contributed methodological know-how. EH carried out the cell culture. SA extracted the total RNA and performed a part of the qPCR analysis. AE re-evaluated the histopathology of tumor samples and evaluated the immunohistochemical staining. KJ supplied the TMA material and performed the histopathological re-evaluation. BC participated in the conception and design of the study, and drafted the manuscript. All authors read and approved the final manuscript.
